# Effects of partial replacement of soybean meal with *Chlorella vulgaris* and lysozyme on diarrheal incidence, plasma biochemical parameters, and immunity of weaned piglets

**DOI:** 10.3389/fvets.2024.1505540

**Published:** 2025-01-13

**Authors:** Md. Abul Kalam Azad, Bowen Li, Ting Ye, Binghua Qin, Qian Zhu, Yordan Martinez, Xiangfeng Kong

**Affiliations:** ^1^CAS Key Laboratory of Agro-Ecological Processes in Subtropical Region, Hunan Provincial Key Laboratory of Animal Nutritional Physiology and Metabolic Process, Institute of Subtropical Agriculture, Chinese Academy of Sciences, Changsha, Hunan, China; ^2^College of Advanced Agricultural Sciences, University of Chinese Academy of Sciences, Beijing, China; ^3^Department of Monogastric Animals, Institute of Animal Science, San José de Las Lajas, Mayabeque, Cuba

**Keywords:** alternative feed, immunity, microalgae, gut health, Xiangcun black pigs

## Abstract

**Introduction:**

The study aimed to investigate the effects of *Chlorella vulgaris* and lysozyme on growth performance, diarrhea rate, immune function, plasma biochemical parameters, and gut microbiota and metabolites of weaned piglets.

**Methods:**

Thirty weaned Xiangcun black piglets (BW, 11.47 ± 1.13 kg) were randomly assigned to one of three treatment groups: corn-soybean meal-based basal diet (CON group), soybean meal replaced with 5% *C. vulgaris* (CHV group), and soybean meal replaced with 5% *C. vulgaris* and 100 mg/kg lysozyme (LYSO group).

**Results:**

Growth performance was not affected by *C. vulgaris* or *C. vulgaris* with lysozyme supplementation, while soybean meal partially replaced by *C. vulgaris* without lysozyme reduced the diarrhea rate of weaned piglets. Plasma biochemical analysis showed that plasma albumin, alkaline phosphatase, and high-density lipoprotein-cholesterol (HDL-C) levels in the CHV group and the total cholesterol and HDL-C levels in the LYSO group were higher when compared with the CON group. The LYSO group had increased interleukin (IL)-10 level in the jejunum and IL-1β level in the ileum while having a decreasing IL-6 level in the jejunum of piglets. Additionally, although Firmicutes and *Megashaera_A* abundances and short-chain fatty acid concentrations (including acetate, propionate, butyrate, and valerate) were reduced in the CHV group, but several beneficial bacteria (such as Actinobacteroita, *Faecealibacterium,* and *Anaerovibrio*) abundances were increased in the LYSO group.

**Discussion:**

In summary, dietary *C. vulgaris* or *C. vulgaris* with lysozyme supplementation improved health of piglets in some contexts without affecting growth performance. Therefore, soybean meal replaced by 5% *C. vulgaris* with or without lysozyme as sustainable feed ingredients in piglet diets could be a viable alternative approach.

## 1 Introduction

China is the largest pig producer in the world, and the pig industry is one of the important sources of the agricultural economy in China. Feed resources for pig production, especially corn and soybeans, are the main raw materials for high-quality feed (1). To meet the high-quality protein requirements for livestock production, China is mostly dependent on imported conventional protein sources crops such as soybeans and other feedstuffs, accounting for more than 80% of the total demands of the country, which limits the sustainable development of the pig industry. Additionally, protein sources from soybeans are also directly in competition with human nutrition. Therefore, the use of non-conventional feed resources as protein alternatives, such as agricultural by-products, insect meals, microalgae, etc., has gained the recent most important global research topic for sustainable animal production.

Microalgae has become one of the most non-conventional feed resources for livestock production due to their faster growth rate, higher biomass productivity and biodiversity, and no competition with other conventional protein resource land requirements (2). Microalgae, multicellular marine microorganisms, have gained more interest in animal nutrition due to their rich composition of essential nutrients, such as crude protein and bioactive compounds (3). *Chlorella vulgaris*, a green microalga, has been found as a promising feed resource for animal production with similar crude protein contents up to 67% in dry matter, with digestibility comparable to the soybean-based meal (3). In addition, *C. vulgaris* is also a rich source of polysaccharides, lipids, minerals, and vitamins (4, 5). In this context, the application of *C. vulgaris* in livestock feed resources would be a potential research target for crucial protein replacement and other bioactive compounds that can enhance animal immune response, disease inhibition, and microbiome colonization (6). However, like other microalgae such as *Arthrospira platensis*, *C. vulgaris* displays several challenges in nutrient availability when included in monogastric diets due to its recalcitrant cell wall. Indeed, several recent studies *in vitro* and *in vivo* have shown that exogenous carbohydrate-active enzymes (CAZymes) could effectively enhance the nutrient utilization of microalgae by monogastric animals (6, 7). For example, Spirulina (*A. platensis*) supplementation with CAZymes (such as lysozyme and Rovabio^®^ Excel AP) increased the dry matter digestibility in weaned piglets without affecting the animal performance compared to the piglets fed without enzyme (8). Therefore, lysozyme, a well-known carbohydrate cell wall degrading enzyme, can break down insoluble mucopolysaccharides into soluble glycopeptides, inducing the damaged cell wall to release its internal contents and easily digested in the gastrointestinal tract of animals (7). However, the underlying mechanism of *C. vulgaris* and lysozyme in the metabolism consequences of pigs still needs to be fully uncovered.

Therefore, we hypothesize that dietary supplementation of *C. vulgaris* and lysozyme could exert beneficial effects in weaned piglets and thus allow them to be utilized as partial replacement for conventional feeding strategies. Thus, the present study aimed to explore the effects of partial replacement of a conventional protein source (soybean meal) from a corn-soybean meal-based diet with 5% *C. vulgaris* with or without lysozyme on growth performance, diarrhea rate, immunity, plasma biochemical parameters, and intestinal microbiota and metabolites in weaned Xiangcun black pigs. The outcomes of this study will provide important guiding significance for the rational use of non-conventional feed resources in pig production.

## 2 Materials and methods

### 2.1 Animals, diets, and management

Thirty weaned Xiangcun black pigs (28 days of age; half male and half female; male piglets were castrated) were selected for the feeding trial. After 7 days of the adaptation period, piglets with an average body weight (BW) of 11.47 ± 1.13 kg were randomly allocated into three groups with 10 pigs (replicates) per group. Three groups included the control group (CON, fed a corn-soybean meal-based basal diet), *C. vulgaris* group (CHV, fed a corn-soybean meal-based basal diet and soybean meal replaced with 5% *C. vulgaris*), and lysozyme group (LYSO, fed a corn-soybean meal-based basal diet and soybean meal replaced with 5% *C. vulgaris* and 100 mg/kg lysozyme). The feeding trial lasted 56 days. Dietary *C. vulgaris* and lysozyme were uniformly mixed with the basal diets. Dietary *C. vulgaris* was obtained from Xian Saiyang Biotechnology Co. Ltd. (Xian, China), and lysozyme was provided by the Sunson Bioscience Technology Development Co. Ltd. (Beijing, China). The supplementing dose of *C. vulgaris* and lysozyme was based on the previous findings (9, 10). The experimental diet’s ingredients and proximal composition were formulated (Table 1) to meet the Chinese nutrient requirements for domestic pigs in China (NY/T65-2004).

**Table 1:** Composition and nutrient levels of the experimental diets (air-dry basis, %).

Items	CON group	CHV group	LYSO group
Ingredients, %			
Corn	61.92	61.97	61.97
Soybean meal	18.50	13.50	13.50
Fish meal	2.00	2.00	2.00
Wheat bran	12.00	11.00	11.00
Soybean oil	2.00	3.00	2.99
*L*-Lysine HCl (78.5%)	0.50	0.40	0.40
*DL*-Methionine (98%)	0.10	0.10	0.10
*L*-Threonine (98.5%)	0.20	0.20	0.20
*L*-Tryptophan (98%)	0.05	0.05	0.05
Di-calcium phosphate	0.75	0.80	0.80
Limestone powder	0.68	0.68	0.68
NaCl	0.30	0.30	0.30
Premix^1^	1.00	1.00	1.00
*Chlorella vulgaris*	0.00	5.00	5.00
Lysozyme	0.00	0.00	0.01
Total	100.00	100.00	100.00
Nutrient levels^2^			
Digestive energy (MJ/kg)	14.29	14.21	14.25
Crude protein	17.00	17.14	16.86
Ether extract	10.77	10.80	9.23
SID Lys	1.14	1.34	1.35
SID Met	0.02	0.01	0.04
SID Thr	0.75	0.85	0.75
SID Trp	0.22	0.33	0.30
Calcium	0.55	0.52	0.59
Total phosphorus	0.61	0.69	0.69

1 Providing the following amounts of vitamins and minerals per kg of a complete diet on as-feed basis: 1800 IU vitamin A, 200 IU vitamin D3, 11 mg vitamin E, 0.5 mg vitamin K, 1 mg vitamin B1, 3.5 mg vitamin B2, 1.5 mg vitamin B6, 17.5 mg vitamin B12, 15 mg niacin, 10 mg pantothenic acid, 0.3 mg folic acid, 0.05 mg biotin, 12 mg CuSO45H2O, 8 mg MnSO4H2O, 150 mg ZnSO4H2O, 170 mg FeSO4H2O, 0.2 mg I, 0.2 mg Se, 0.2 mg Co.

2 Nutrient levels are measured values in triplicate.

Experimental pigs were housed in individual pens (1.10 × 0.60 m) equipped with an adjusted drinking water nipple, single-hole feeder, and plastic-slatted floored room. The piggery was facilitated with forced ventilation and environmentally controlled temperature (23–26°C) and humidity (60 ± 5%). All pigs had free access to water and food at all times. All experimental animals enrolled in this study were in good health conditions, and had no antibiotic exposure or any gastrointestinal diseases before the trial.

### 2.2 Sample collection and processing

At the end of the feeding trial and 12 h after fasting, all pigs were selected for sampling after weighing. Blood samples (10 mL) were collected from the anterior vena cava of each pig into anticoagulated vacuum blood collection tubes, mixed upside down, and then centrifuged for 10 min at 3,500 × *g* and 4°C to obtain the plasma. The plasma samples were immediately stored at −80°C for further analyses of immunoglobulins and immuno-cytokines. Afterward, all pigs were anesthetized with an intramuscular injection of Zoletil^®^ 50 (Tiletamine and zolazepam; Beijing Lab Anim. Tech. Dev. Co. Ltd., Beijing, China) and exsanguinated to collect jejunum, ileum, and colon samples. The samples (2–3 cm) of the jejunum (10 cm below the flexure of duodenum-jejunum) and ileum (10 cm above the ileo-cecal junction) were excised, flushed in phosphate buffer solution, and then scrapped by glass slides. The mucosa scrapings (~2 g) were sampled, snap-frozen in liquid nitrogen, and immediately stored at −80°C to determine intestinal immuno-cytokines. Colonic contents (mid-section) were harvested into 1.5 mL sterile tubes and stored at −80°C for colonic microbiota and metabolite analyses.

### 2.3 Determination of growth performance and diarrhea rate

The initial and final BW of each pig was measured to calculate the average daily gain (ADG). Feed consumption and feed refusal of each pig were recorded daily to calculate the average daily feed intake (ADFI) of pigs. The ratio of feed intake to weight gain (F/G) was calculated. The diarrhea rate of experimental pigs was evaluated daily using Hart and Dobb’s method as described previously (11, 12). Briefly, the diarrheal conditions of pigs were recorded daily as a fecal score of 0 for normal, 1 for mild diarrhea, 2 for moderate diarrhea, and 3 for severe diarrhea, respectively. The diarrhea rate (%) was calculated as follows:

Total diarrhea times/(total number of piglets × experimental days) × 100.

### 2.4 Determination of plasma biochemical parameters and immunoglobulins

Plasma biochemical parameters, including total protein (TP), albumin (ALB), ammonia (AMM), triglycerides (TG), total cholesterol (TC), high-density lipoprotein-cholesterol (HDL-C), low-density lipoprotein-cholesterol (LDL-C), and glucose (GLU) concentrations, as well as alpha-amylase (*α*-AMY), alanine aminotransferase (ALT), aspartate aminotransferase (AST), alkaline phosphatase (ALP), lactate dehydrogenase (LDH), and cholinesterase (CHE) activities were analyzed using their corresponding available kits (F. Hoffmann-La Roche Ltd., Basel, Switzerland) and instruments (Cobas c311, Basel, Switzerland). The levels of plasma immunoglobulins, including immunoglobulin A (IgA, RC-01340P2), IgG (RC-01237P2), and IgM (RC-01236P2) were determined using the commercially available ELISA kits (Haiyang Biological Co. Ltd., Changsha, China) and following the guidelines provided by the manufacturer.

### 2.5 Analysis of plasma and intestinal immuno-cytokines

The concentrations of plasma, jejunal, and ileal interleukin (IL)-1β (RC-01256P1), IL-2 (RC-01255P1), IL-6 (RC-01252P1), IL-10 (RC-01255P1), IL-17 (RC-01211P1), tumor necrosis factor (TNF)-*α* (RC-01217P1), and interferon (IFN)-*γ* (RC-01246P1) were evaluated with commercially available porcine-specific ELISA kits (Haiyang Biological Co. Ltd., Changsha, China) according the guidelines provided by the company. Absorbance values were read on a Multiscan Spectrophotometer (Infinite M200 PRO; TECAN, Männedorf, Switzerland).

### 2.6 Colonic microbial DNA extraction, 16S rRNA pyrosequencing, and bioinformatics analysis

Approximately 0.30 g of colonic contents of each sample were thawed and homogeneously mixed for bacterial genomic DNA extraction with a Mag-Bind^®^ Stool DNA kit (Omega, Guangzhou, China). The concentration and quality of the extracted DNA were determined with the NanoDrop OneC miniature Volume UV–Vis spectrophotometer (Thermo Fisher Scientific, Waltham, MA, USA), and agarose gel electrophoresis was used for resulting PCR products. The hypervariable V4–V5 region of the colonic bacterial 16S rRNA genes was amplified with universal forward primer 338F (5′-ACTCCTACGGGGAGGCAGCA-3′) and reverse primer 806R (5′-GGACTACHVGGGGTWTCTAAT-3′). The PCR reaction thermal cycle conditions were followed by the manufacturer’s standard protocols (New England Biolab. Inc., Ipswich, MA, USA). The obtained PCR products were purified with Vazyme VAHTSTM DNA Clean Beads to remove non-specific products, and then the constructed DNA libraries were quantitatively analyzed using the Quant-iT PicoGreen dsDNA Detection Kit (Invitrogen, Carlsbad, CA, USA). Based on the standard protocols of Shanghai Personal Biotech. Co. Ltd. (Shanghai, China), purified amplicons were subjected to equimolar and pair-end (2 × 300) sequencing by the MiSeq Reagent kit on an Illumina MiSeq platform (Illumina, San Diego, CA, USA).

The raw sequence data obtained from the Illumina Miseq were processed using the DADA2 plugin and QIIME2 for sequence and subsequent data analysis as previously described (13). After that, using the default parameter, a representative sequence was selected from each operational taxonomic unit (OTU) and classified based on BLAST searching against the Greengenes database. The alpha-diversity indices, including Chao 1, Shannon index, Simpson index, and Observed_species, were calculated using the OTU table and QIIM2. Beta-diversity analysis was performed using principal coordinate analysis (PCoA) and non-measured multidimensional scaling (NMDS) plots based on the Bray-Curtis distance metric to identify the structural differences in microbial communities among samples. The differences in the taxonomic composition of colonic microbiota at the phylum and genus levels were evaluated using the Kruskal-Wallis test. The linear discriminant analysis size effect (LEfSe) was performed to analyze the taxonomic hierarchical distribution of marker species in each group of samples using the histograms of the distribution of linear regression analysis (LDA) values of different species and species taxonomic branching diagrams (Cladograms) to present the significantly enriched species and their levels of importance in each group of samples.

### 2.7 Determination of colonic microbial metabolites

The short-chain fatty acid (SCFA), including straight-chain fatty acids (acetate, propionate, butyrate, and valerate) and branched-chain fatty acids (isobutyrate and isovalerate) concentrations in the colonic contents of weaned pigs were measured with the gas chromatography (7890A; Agilent Technologies Inc., Santa Clara, CA, USA), following the previously described protocols (14). Briefly, colonic contents (1.00 g) were homogeneously mixed with ultrapure water (5 mL), and supernatants were obtained by centrifuging at 1000 × *g* and 4°C for 10 min. The obtained supernatants were mixed with 25% metaphosphoric acid at a 9:1 (v/v) ratio in 2 mL sterile centrifuge tubes, centrifuged at 2000 × *g* and 4°C for 10 min, and then filtered through a 0.45-μm polysulfone filter for gas chromatography analysis.

### 2.8 Statistical analysis

Data analyses of growth performance, plasma biochemical parameters, immunoglobulins, and plasma and intestinal immuno-cytokines were analyzed by one-way analysis of variance (ANOVA). Multiple comparisons among different groups were evaluated using Tukey’s *post-hoc* test with the SPSS 26.0 software package (IBM Inc., Chicago, IL, USA). In addition, two pigs were removed from the microbiome analysis due to experimental outliers, and the sample size was adjusted to *n* = 8 per group, as indicated where applicable. Colonic microbiome data were analyzed using the Kruskal-Wallis test. The individual pigs were considered the experimental unit for all analyses. All data are expressed as means with their pooled standard error of the means (SEM). Differences among groups were considered statistically significant when *p* < 0.05, and a trend at 0.05 ≤ *p* < 0.10. Correlations between abundant top 20 bacterial taxa at the genus level and plasma biochemical parameters, immunoglobulins, immuno-cytokines, and colonic SCFA concentrations were analyzed using Spearman’s rank correlation test.

## 3 Results

### 3.1 Effects of *Chlorella vulgaris* and lysozyme on the growth performance and diarrhea rate of weaned pigs

The effects of dietary *C. vulgaris* and lysozyme supplementation on the growth performance of weaned Xiangcun black pigs are presented in Table 2. The diarrhea rate was decreased (*p* < 0.001) in the CHV group compared with the CON and LYSO groups. Additionally, the diarrhea rate in the LYSO group was increased regarding the CON and CHV groups (*p* < 0.001). However, dietary *C. vulgaris* and lysozyme had no significant impacts (*p* > 0.10) on the growth performance parameters, including BW, ADG, ADFI, and F/G of weaned pigs.

**Table 2:** Effects of *C. vulgaris* and lysozyme on the growth performance and diarrhea rate of weaned pigs.

Item	CON	CHV	LYSO	SEM	*p*-values
Initial BW (kg)	11.42	11.52	11.49	0.22	0.984
Final BW (kg)	32.11	33.20	31.38	0.65	0.539
ADG (g/d)	389.68	376.88	356.15	12.60	0.567
ADFI (g/d)	1198.03	1201.10	1236.82	29.84	0.851
F/G	3.13	3.21	3.53	0.10	0.211
Diarrhea rate (%)	4.82^b^	2.44^c^	7.14^a^	0.39	<0.001

Data are expressed as means with pooled SEM (*n* = 9–10). Mean values within the same row without a common superscript letter differ significantly (*p* < 0.05). CON, pigs fed a corn-soybean meal-based basal diet; CHV, pigs fed a corn-soybean meal-based basal diet and soybean meal replaced with 5% *C. vulgaris*; LYSO, pigs fed a corn-soybean meal-based basal diet and soybean meal replaced with 5% *C. vulgaris* and 100 mg/kg lysozyme; ADG, average daily gain; ADFI, average daily feed intake; BW, body weight; F/G, feed to gain ratio.

### 3.2 Effects of *Chlorella vulgaris* and lysozyme on the plasma biochemical parameters of weaned pigs

The changes in plasma biochemical parameters of weaned Xiangcun black pigs are listed in Table 3. The plasma ALB (*p* = 0.052) concentration and ALP (*p* = 0.090) activity displayed increasing trends in the CHV group compared with the CON and LYSO groups. The plasma TC concentration was higher (*p* < 0.05) in the LYSO group compared with the CON and CHV groups. Moreover, plasma HDL-C concentration was higher (*p* < 0.05) in the CHV and LYSO groups compared with the CON group. No significant differences (*p* > 0.10) were observed in other plasma biochemical parameters among the three groups.

**Table 3:** Effects of *C. vulgaris* and lysozyme on the plasma biochemical parameters and immunoglobulins of weaned piglets.

Item	CON	CHV	LYSO	SEM	*p*-values
TP (g/L)	67.21	68.63	67.96	0.64	0.724
ALB (g/L)	37.75^y^	43.41^x^	38.98^y^	1.02	0.052
ALT (U/L)	42.89	47.35	47.54	2.48	0.705
AST (U/L)	52.75	53.88	49.25	2.81	0.797
ALP (U/L)	143.13^y^	207.50^x^	157.50^y^	12.70	0.090
LDH (U/L)	537.50	565.88	551.25	11.43	0.619
GLU (mmol/L)	7.34	6.74	7.38	0.31	0.660
TG (mmol/L)	0.62	0.49	0.58	0.04	0.479
TC (mmol/L)	2.09^b^	2.16^b^	2.43^a^	0.06	0.033
HDL-C (mmol/L)	0.97^b^	1.10^a^	1.19^a^	0.03	0.005
LDL-C (mmol/L)	1.15	1.05	1.21	0.04	0.347
CHE (μmol/L)	682.63	782.25	746.38	25.74	0.289
AMM (μmol/L)	100.48	111.10	116.33	12.12	0.873
IgA (g/L)	17.27	16.37	17.99	0.50	0.431
IgG (g/L)	16.50	16.05	16.56	0.17	0.423
IgM (g/L)	16.46^a^	14.74^b^	15.80^a^	0.24	0.002

Data are expressed as means with pooled SEM (*n* = 9–10). ^a,b^ Mean values within the same row without a common superscript letter differ significantly at *P* < 0.05; and ^x,y^ mean values without a common superscript letter displayed tendency at 0.05 ≤ *P* < 0.10. CON, pigs fed a corn-soybean meal-based basal diet; CHV, pigs fed a corn-soybean meal-based basal diet and soybean meal replaced with 5% *C. vulgaris*; LYSO, pigs fed a corn-soybean meal-based basal diet and soybean meal replaced with 5% *C. vulgaris* and 100 mg/kg lysozyme; AMM, ammonia; ALB, albumin; ALP, alkaline phosphatase; ALT, alanine aminotransferase; AST, aspartate aminotransferase; CHE, cholinesterase; GLU, glucose; HDL-C, high-density lipoprotein-cholesterol; Ig, immunoglobulin; LDH, lactate dehydrogenase; LDL-C, low-density lipoprotein-cholesterol; TC, total cholesterol; TG, triglyceride; TP, total protein.

### 3.3 Effects of *Chlorella vulgaris* and lysozyme on the immune function of weaned pigs

The level of plasma IgM was decreased (*p* < 0.05) in the CHV group compared with the CON and LYSO groups. However, supplementation of *C. vulgaris* and lysozyme did not affect (*p* > 0.10) the plasma levels of IgA and IgG in weaned pigs (Table 3).

As shown in Table 4, the IL-6 content was lower while IL-10 content was higher in the jejunum of the LYSO group (*p* < 0.05) compared with the CON and CHV groups. In the ileum, the IL-1β content was higher (*p* < 0.05) in the LYSO group compared with the CON group. Moreover, the TNF-*α* content was lower (*p* < 0.05) in the ileum of the CHV group compared with the CON and LYSO groups. However, supplementation of *C. vulgaris* and lysozyme did not affect (*p* > 0.10) the plasma immno-cytokines levels.

**Table 4:** Effects of *C. vulgaris* and lysozyme on plasma and intestinal immuno-cytokine levels of weaned piglets (pg/mL).

Item	CON	CHV	LYSO	SEM	*p*-values
Plasma					
IL-1β	158.27	153.98	144.19	3.47	0.234
IL-2	112.58	107.38	104.28	5.01	0.811
IL-6	21.38	19.91	20.28	0.31	0.138
IL-10	39.19	41.90	42.14	1.19	0.545
IL-17	23.69	22.20	24.37	0.50	0.218
TNF-α	459.15	460.44	496.32	14.50	0.548
IFN-γ	438.62	467.86	406.16	12.59	0.133
Jejunum					
IL-1β	151.49	145.57	149.10	1.90	0.463
IL-2	109.06	104.79	115.17	2.14	0.136
IL-6	18.03^a^	17.91^a^	16.68^b^	0.24	0.029
IL-10	41.60^b^	41.15^b^	45.39^a^	0.80	0.049
IL-17	27.08	24.88	24.30	1.35	0.699
TNF-α	566.77	541.91	537.79	7.29	0.204
IFN-γ	451.64	407.05	447.14	10.78	0.166
Ileum					
IL-1β	152.34^b^	161.30^ab^	170.00^a^	2.50	0.007
IL-2	110.77	110.02	104.22	2.46	0.520
IL-6	15.78	15.06	15.59	0.35	0.711
IL-10	44.82	41.08	41.31	0.91	0.198
IL-17	23.99	25.88	28.42	0.85	0.113
TNF-α	574.35^a^	486.72^b^	593.70^a^	16.64	0.007
IFN-γ	449.22	465.72	495.07	10.39	0.196

Data are expressed as means with pooled SEM (*n* = 9–10). ^a,b^ Mean values within the same row without a common superscript letter differ significantly at *p* < 0.05. CON, pigs fed a corn-soybean meal-based basal diet; CHV, pigs fed a corn-soybean meal-based basal diet and soybean meal replaced with 5% *C. vulgaris*; LYSO, pigs fed a corn-soybean meal-based basal diet and soybean meal replaced with 5% *C. vulgaris* and 100 mg/kg lysozyme; IFN, interferon; IL, interleukin; TNF, tumor necrosis factor.

### 3.4 Effects of *Chlorella vulgaris* and lysozyme on the colonic microbiota diversity of weaned pigs

**Figure 1:**
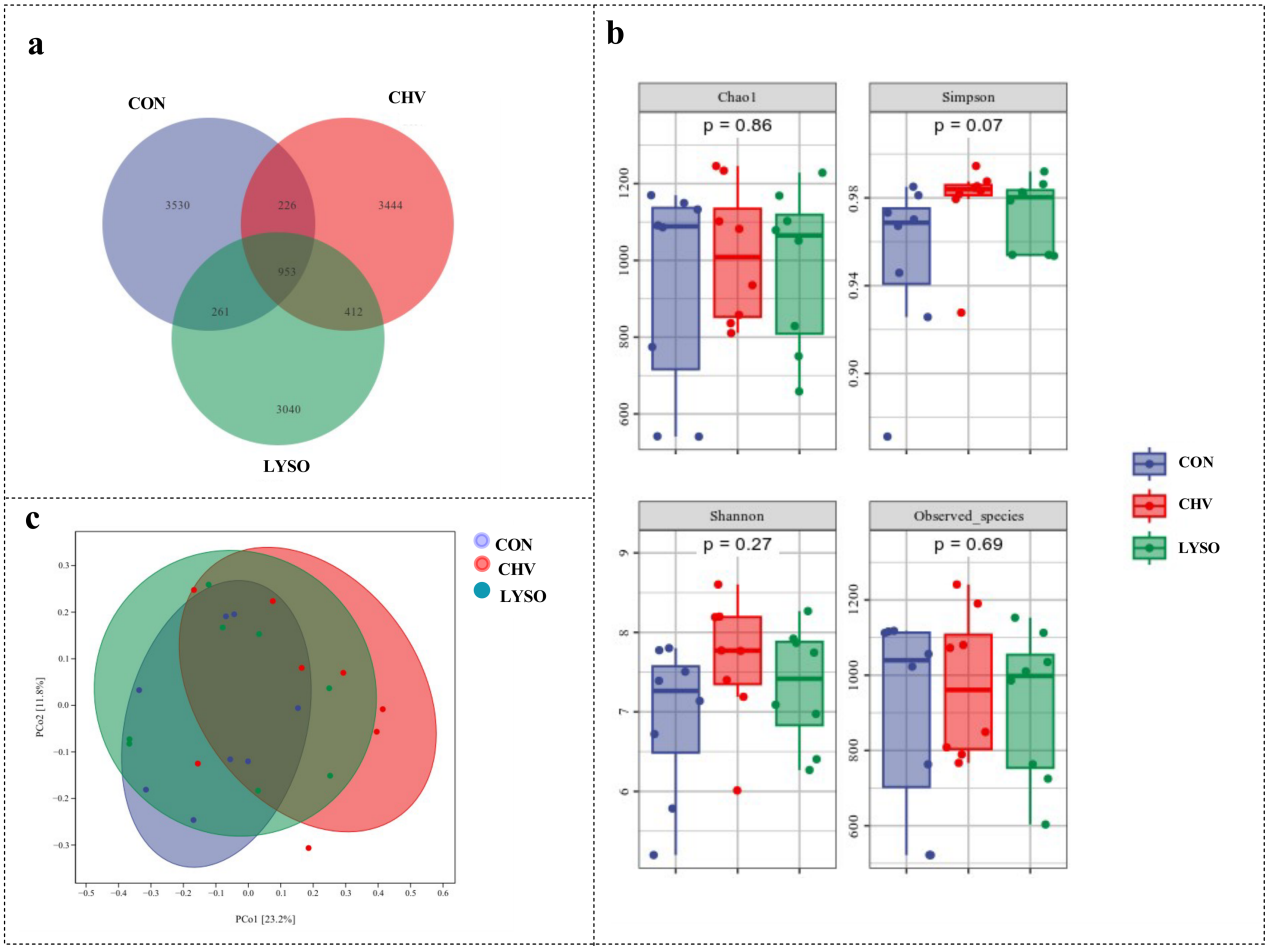
Effects of *C. vulgaris* and lysozyme on colonic microbiota diversity (*n* = 8). **(a)** The Venn, **(b)** alpha-diversity, and **(c)** principal coordinate analyses. Each dot symbol represents the gut microbiota of each piglet. CON, pigs fed a corn-soybean meal-based basal diet; CHV, pigs fed a corn-soybean meal-based basal diet and soybean meal replaced with 5% *C. vulgaris*; LYSO, pigs fed a corn-soybean meal-based basal diet and soybean meal replaced with 5% *C. vulgaris* and 100 mg/kg lysozyme.

Colonic contents from 24 samples (*n* = 8) were assessed to evaluate the effects of *C. vulgaris* and lysozyme on the microbiota diversity of weaned pigs. The Venn analysis (Figure 1a) showed that there were 3,530, 3,444, and 3,040 unique OTUs in the colonic contents of the CON, CHV, and LYSO groups, respectively. Among them, there were 953 common OTUs among the three groups, of which 226, 261, and 412 common OTUs between the CON vs. CHV, CON vs. LYSO, and CHV and LYSO groups, respectively (Figure 1a).

The alpha-diversity analysis was performed to identify the diversity and structure of the colonic microbiota among different groups. The Simpson index displayed an increasing trend (*p* = 0.07) in the CHV group compared with the CON and LYSO groups. However, there were no significant changes (*p* > 0.10) in the Chao1, Shannon, and Observed_species among the three groups (Figure 1b). Furthermore, the beta-diversity analysis was performed to visualize the differences in the colonic microbiota community among the three groups. However, the PCoA showed no distinct separation among the three groups (Figure 1c).

### 3.5 Effects of *Chlorella vulgaris* and lysozyme on the colonic microbiota structure of weaned pigs

**Figure 2:**
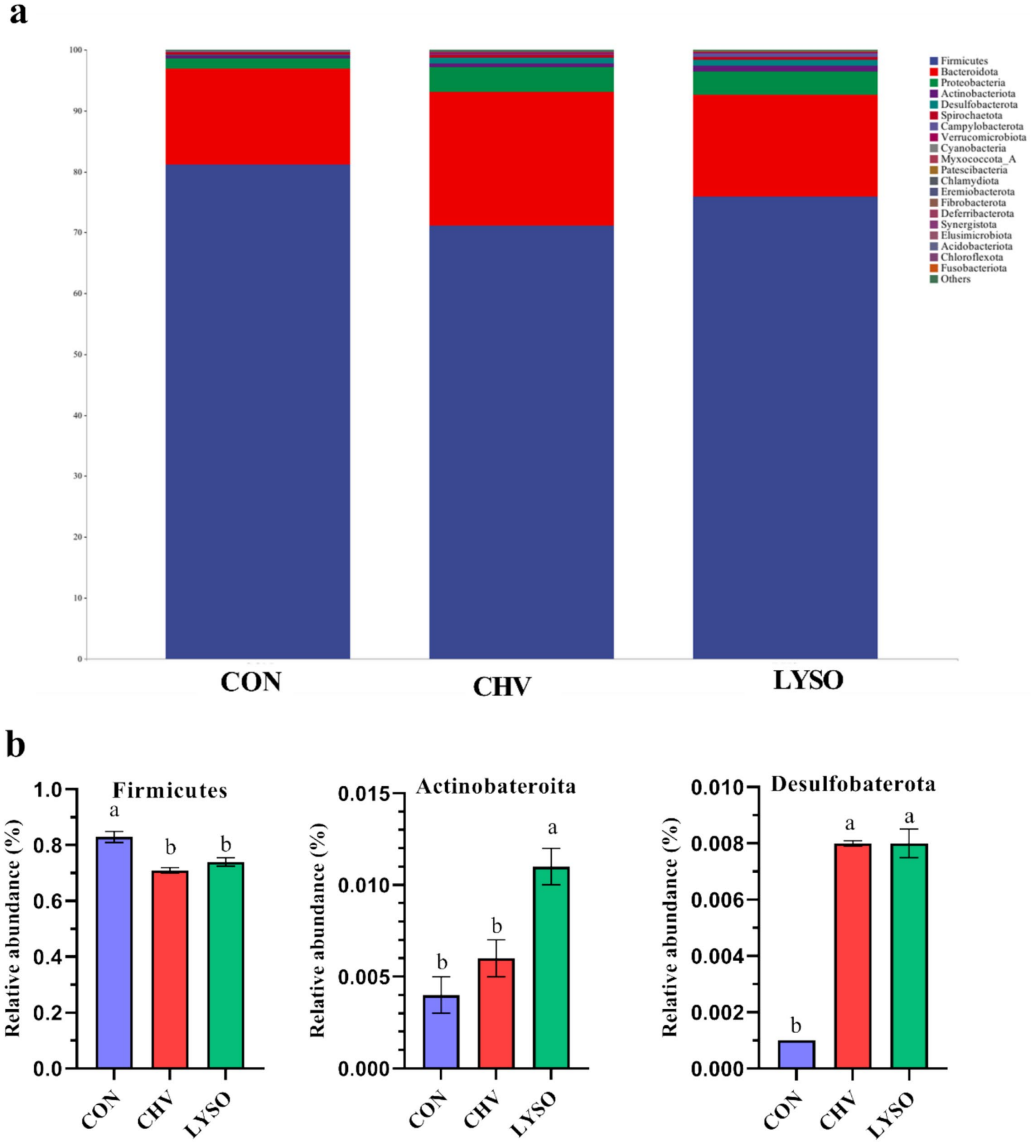
Effects of *C. vulgaris* and lysozyme on colonic microbiota composition **(a)** and taxonomic differences **(b)** at the phylum level (*n* = 8). Bars without a common letter indicate a significant difference (*p* < 0.05). CON, pigs fed a corn-soybean meal-based basal diet; CHV, pigs fed a corn-soybean meal-based basal diet and soybean meal replaced with 5% *C. vulgaris*; LYSO, pigs fed a corn-soybean meal-based basal diet and soybean meal replaced with 5% *C. vulgaris* and 100 mg/kg lysozyme.

At the phylum level, Firmicutes, Bacteroidota, and Proteobacteria were the top three bacterial phyla (>90%) in the colon of weaned pigs in all groups, while other phyla were shown at very low relative abundance (Figure 2a). The top three phyla (including Firmicutes, Bacteroidota, and Proteobacteria) accounted for 81.16, 15.74, and 1.69% in the CON group, 71.16, 21.88, and 4.11% in the CHV group, and 75.90, 16.66, and 3.82% in the LYSO group, respectively (Figure 2a). The relative abundance of Firmicutes was decreased, while Desulfobacterota was increased in the CHV and LYSO groups compared with the CON group (*p* < 0.05). Moreover, the relative abundance of Actinobacteriota was higher (*p* < 0.05) in the LYSO group compared with the CON and CHV groups (Figure 2b).

**Figure 3:**
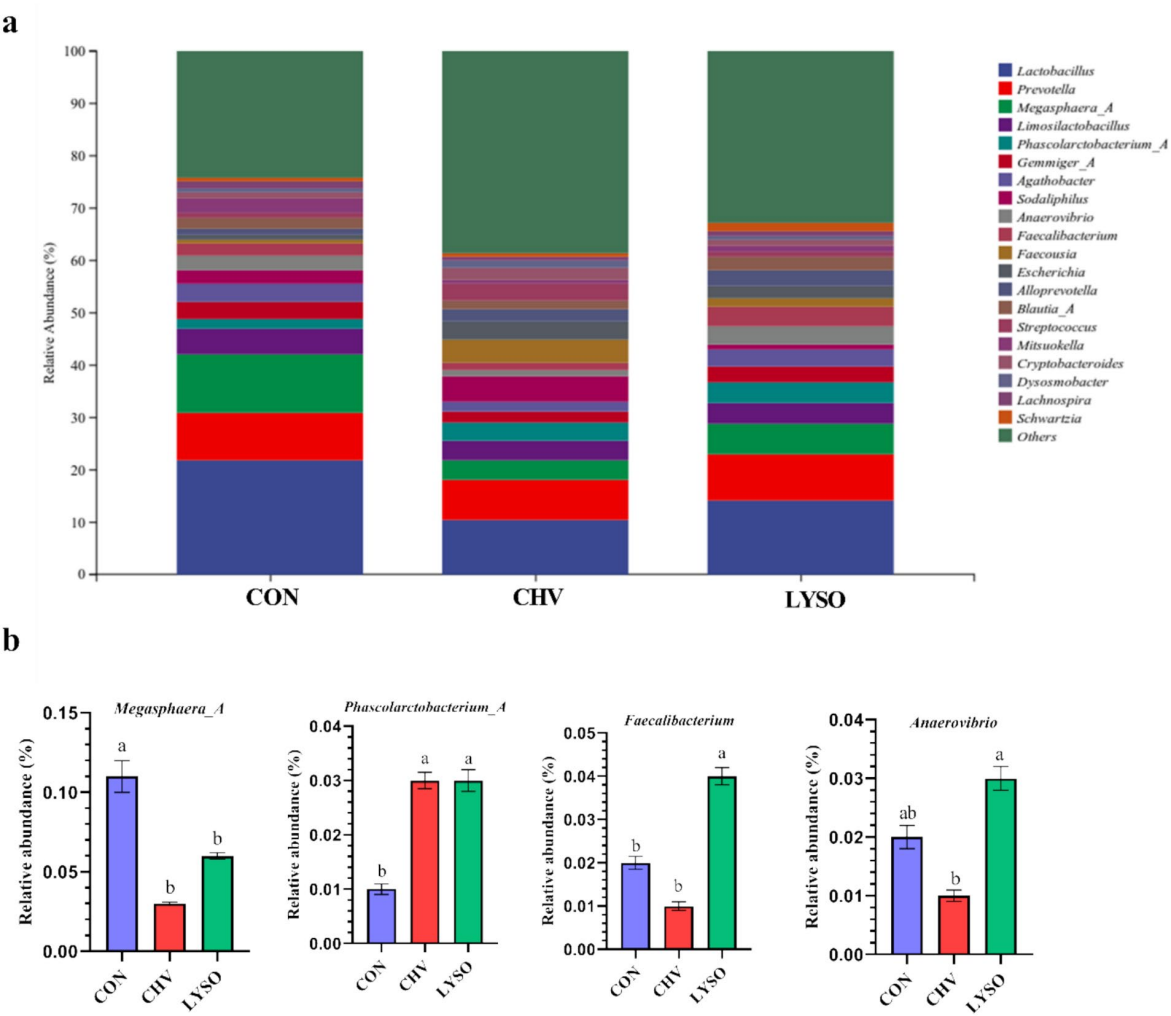
Effects of *C. vulgaris* and lysozyme on colonic microbiota composition **(a)** and taxonomic differences **(b)** at the genus level (*n* = 8). Bars without a common letter indicate a significant difference (*p* < 0.05). CON, pigs fed a corn-soybean meal-based basal diet; CHV, pigs fed a corn-soybean meal-based basal diet and soybean meal replaced with 5% *C. vulgaris*; LYSO, pigs fed a corn-soybean meal-based basal diet and soybean meal replaced with 5% *C. vulgaris* and 100 mg/kg lysozyme.

Colonic bacterial community composition at the genus level is shown in Figure 3. *Lactobacillus*, *Prevotella*, and *Megasphaera_A* were the top three bacterial genera in the colon of all pigs. *Lactobacillus* (21.71%), *Megasphaera_A* (11.25%), *Prevotella* (9.10%), *Limosilactobacillus* (4.88%), *Gemmiger_A* (3.43%), *Agathobacter* (3.47%), *Faecalibacterium* (2.28%), and *Phascolarctobacterium_A* (1.74%) were the most abundant bacterial genera in the CON group. The most abundant bacterial genera in the CHV group were *Lactobacillus* (10.27%), *Prevotella* (7.75%), *Sodaliphilus* (4.87%), *Limosilactobacillus* (3.85%), *Megasphaera_A* (3.72%), *Phascolarctobacterium_A* (3.50%), and *Faecalibacterium* (1.45%). In addition, *Lactobacillus* (10.72%), *Prevotella* (7.75%), *Megasphaera_A* (5.99%), *Limiosilactobacillus* (3.90%), and *Phascolarctobacterium_A* (3.50%) were the most abundant bacterial genera in the LYSO group (Figure 3a). The relative abundance of *Phascolarctobacterium_A* was higher, whereas *Megasphaera_A* was lower in the CHV and LYSO groups compared with the CON group (*p* < 0.05). The relative abundance of *Faecalibacterium* was higher (*p* < 0.05) in the LYSO group compared with the CON and CHV groups, while *Anaerovibrio* was higher (*p* < 0.05) in the LYSO group compared with the CHV group (Figure 3b).

**Figure 4:**
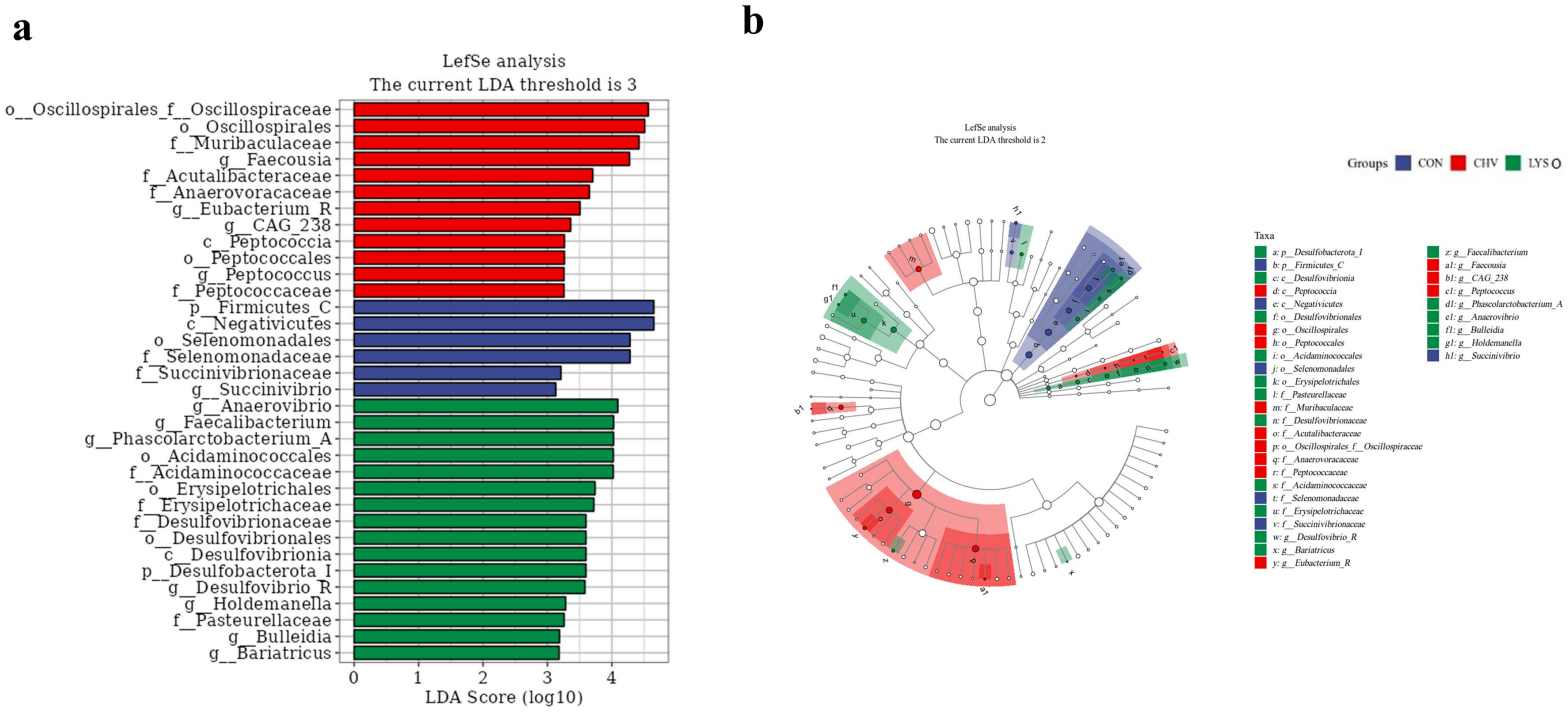
Linear discriminant analysis combined size effects (LEfSe) histogram **(a)** and species taxonomic branching diagram **(b)** of the colonic microbiota of weaned pigs. CON, pigs fed a corn-soybean meal-based basal diet; CHV, pigs fed a corn-soybean meal-based basal diet and soybean meal replaced with 5% *C. vulgaris*; LYSO, pigs fed a corn-soybean meal-based basal diet and soybean meal replaced with 5% *C. vulgaris* and 100 mg/kg lysozyme.

To identify the differences in colonic microbial function among different groups, LEfSe analysis (LDA threshold score ≥ 3.0) was performed at the phylum and genus levels (Figure 4a). There was a significant enrichment of Firmicutes in the CON group and Desulfobacterota in the LYSO group at the phylum level. At the genus level, *Succinivibrio* was enriched in the CON group, while *Eubacterium*, *Faecousia, CAG_238*, and *Peptococcus* were enriched in the CHV group. Additionally, *Dusulfovibrio_R*, *Bariatricus*, *Facccalibacterium*, *Phascolarctobacterium_A*, *Anaerovibrio*, *Bulleidia,* and *Holdemanella* were enriched in the LYSO group (Figure 4a). Moreover, Firmicutes_C in the CON group, *Peptococcia* in the CHV group, and Desulfobacterota_I in the LYSO group were the most dominant microbiota (Figure 4b).

### 3.6 Effects of *Chlorella vulgaris* and lysozyme on the colonic short-chain fatty acid concentrations of weaned pigs

The effects of dietary *C. vulgaris* and lysozyme supplementation on the colonic SCFA concentrations of weaned pigs are presented in Table 5. Colonic acetate, propionate, butyrate, and valerate concentrations were lower (*p* < 0.001) in the CHV and LYSO groups compared with the CON group. However, acetate and butyrate concentrations were higher (*p* < 0.001) in the LYSO group compared with the CHV group. Moreover, colonic isobutyrate and isovalerate concentrations were higher (*p* < 0.05) in the CHV group compared with the CON and LYSO groups.

**Table 5:** Effects of *C. vulgaris* and lysozyme on the colonic short-chain fatty acid concentrations of weaned pigs (μmol/g).

Items	CON	CHV	LYSO	SEM	*p*-values
Acetate	4137.67^a^	2932.28^c^	3382.02^b^	121.64	<0.001
Propionate	2426.91^a^	1245.76^b^	1650.49^b^	124.44	<0.001
Isobutyrate	52.42^b^	84.26^a^	45.48^b^	6.53	0.029
Butyrate	1271.35^a^	708.98^c^	927.26^b^	55.14	<0.001
Isovalerate	92.09^b^	150.65^a^	80.83^b^	11.49	0.023
Valerate	306.44^a^	176.65^b^	185.52^b^	14.75	<0.001

Data are expressed as means with pooled SEM (*n* = 9–10). ^a,b^ Mean values within the same row without a common superscript letter differ significantly at *p* < 0.05. CON, pigs fed a corn-soybean meal-based basal diet; CHV, pigs fed a corn-soybean meal-based basal diet and soybean meal replaced with 5% *C. vulgaris*; LYSO, pigs fed a corn-soybean meal-based basal diet and soybean meal replaced with 5% *C. vulgaris* and 100 mg/kg lysozyme.

### 3.7 Correlations between the levels of plasma biochemical parameters, immunoglobulins, immuno-cytokines, colonic SCFA, and microbiota abundances

**Figure 5:**
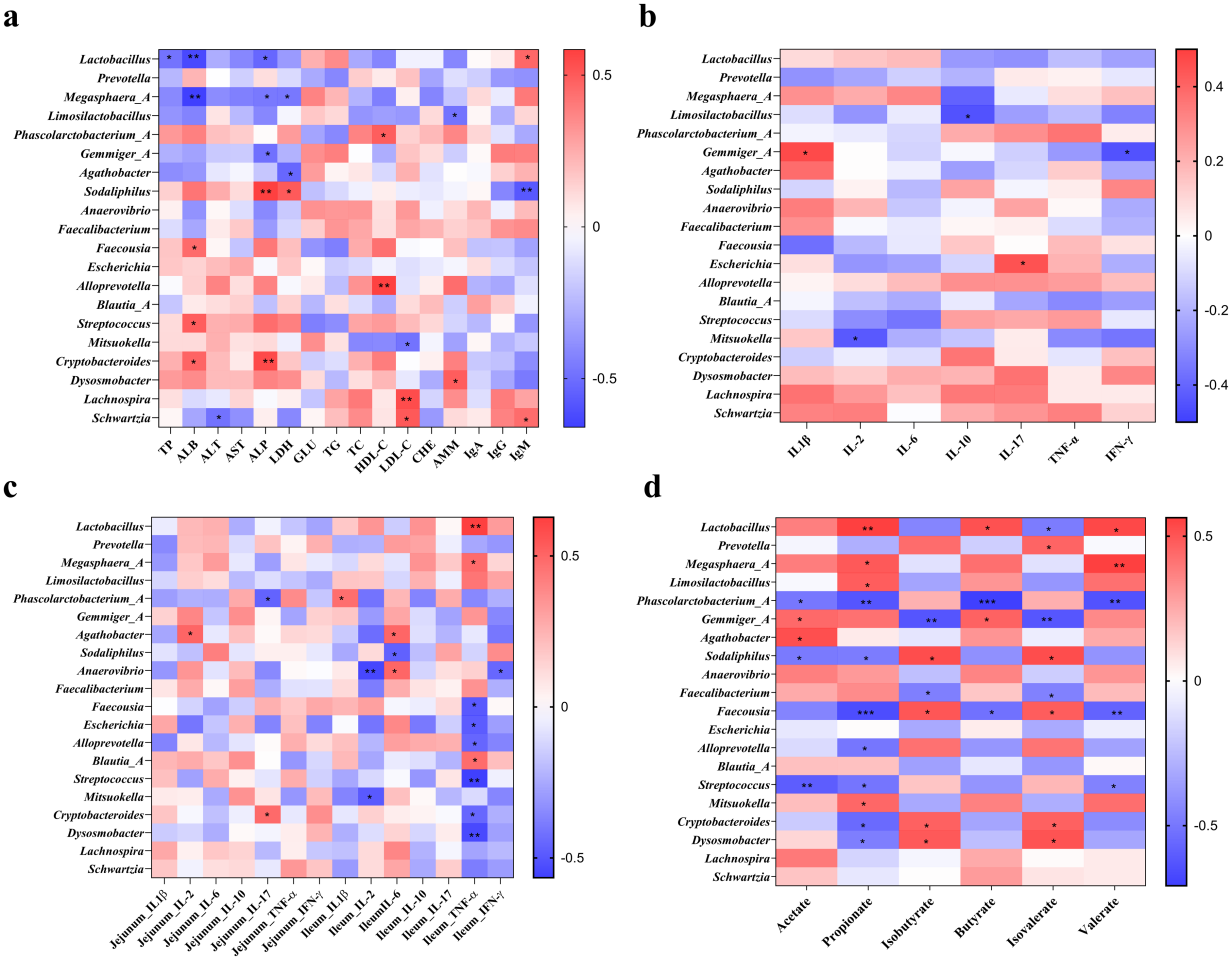
Spearman’s correlation analysis of different colonic microbiota and plasma biochemical parameters and immunoglobulins **(a)**, plasma cytokines **(b)**, intestinal cytokines **(c)**, and colonic metabolites **(d)**. * *p* < 0.05, ** *p* < 0.01, and *** *p* < 0.001.

Spearman’s correlation analysis was performed to reveal the associations between the levels of plasma biochemical parameters, immunoglobulins, immuno-cytokines, colonic SCFA, and the top 20 colonic bacterial taxa at the genus level (Figure 5). Plasma TP was negatively (*p* < 0.05) correlated with *Lactobacillus*, whereas plasma ALB was negatively correlated (*p* < 0.01) with *Lactobacillus* and *Megasphaera_A* and positively correlated (*p* < 0.05) with *Faceousia*, *Streptococcus*, and *Cryptobacteroides*. Plasma ALP was negatively correlated (*p* < 0.05) with *Lactobacillus, Megasphaera_A*, and *Gemmiger_A*, while positively correlated (*p* < 0.01) with *Sodaliphilus* and *Cryptobacteroides*. Plasma LDH was positively correlated with *Sodaliphilus*, while negatively correlated with *Megasphaera_A* and *Agathobacter* (*p* < 0.05). There were positive correlations between plasma HDL-C with *Phascoloractobacterium_A* and *Alloprevotella* and plasma LDL-C with *Lachnospira* and *Schwartzia* (*p* < 0.05). Plasma AMM was positively correlated with *Dysosmobacter* and negatively correlated with *Limosilactobacillus* (*p* < 0.05). Moreover, plasma IgM was positively correlated (*p* < 0.05) with *Lactobacillus* and *Schwartzia* and negatively correlated (*p* < 0.01) with *Sodaliphilus* (Figure 5a).

The correlations between plasma and intestinal immuno-cytokines and colonic bacterial taxa at the genus level are shown in Figures 5b,c. The positive correlation included between plasma IL-1β with *Germmiger_A* and plasma IL-17 with *Escherichia*, while the negative correlation included between plasma IL-2 with *Mitsuokella*, plasma IL-10 with *Limosilactobacillus,* and plasma IFN-*γ* with *Germmiger_A* (Figure 5b). The IL-2 in the jejunum was positively correlated with *Agathobacter*, while IL-17 in the jejunum was positively correlated with *Cryptobacteroides* and negatively correlated with *Phascoloractobacterium_A* (*p* < 0.05). In addition, IL-1β in the ileum was positively correlated with *Phascoloractobacterium_A*, while IL-2 in the ileum was negatively correlated with *Anaerovibrio* and *Mitsuokella* (*p* < 0.05). The TNF-*α* in the ileum was positively correlated with *Lactobacillus*, *Megasphaera*_*A*, and *Blautia_A*, while it was negatively correlated with *Faecousia*, *Escherichia*, *Alloprevotella*, *Streptococcus*, *Cryptobacteroides*, and *Dysosmobacter* (*p* < 0.05). Furthermore, IFN-γ in the ileum was negatively correlated (*p* < 0.05) with *Alloprevotella* (Figure 5c).

The correlations between SCFA and colonic bacterial taxa at the genus level are shown in Figure 5d. The positive correlation included between acetate with *Gemmiger_A* and *Agathobacter* (*p* < 0.05), propionate with *Lactobacillus* (*p* < 0.01), *Megasphaera_A (p < 0.05), Limosilactobacillus (p < 0.05),*, and *Mitsuokella* (*p* < 0.05), butyrate with *Lactobacillus* and *Gemmiger_A* (*p* < 0.05), valerate with *Lactobacillus* (*p* < 0.05) and *Megasphaera*_*A* (*p* < 0.01), isobutyrate with *Sodaliphilus*, *Faecousia*, *Cryptobacteroides*, and *Dysosmobacter* (*p* < 0.05), and isovalerate with *Prevotella*, *Sodaliphilus*, *Faecousia*, *Cryptobacteroides*, and *Dysosmobacter* (*p* < 0.05). The negative correlation (*p* < 0.05) included between acetate with *Phascoloractobacterium_A*, *Sodaliphilus*, and *Streptococcus*, propionate (*p* < 0.05) with *Phascoloractobacterium_A*, *Sodaliphilus*, *Faecousia* (*p* < 0.001), *Alloprevotella*, *Streptococcus*, *Cryptobacteroides*, and *Dysosmobacter*, butyrate with *Phascoloractobacterium_A* (*p* < 0.001) and *Faecousia* (*p* < 0.05), valerate (*p* < 0.01) with *Phascoloractobacterium_A*, *Faecousia*, and *Streptococcus* (*p* < 0.05), isobutyrate with *Germmiger_A* (*p* < 0.01) and *Faecalibacterium* (*p* < 0.05), isovalerate with *Lactobacillus* (*p* < 0.05), *Germmiger_A* (*p* < 0.01), and *Faecalibacterium* (*p* < 0.05).

## 4 Discussion

Feed resources play indispensable roles in sustainable livestock production. Growing demands for protein from similar agricultural sources for both humans and animals are becoming more competitive due to the constantly increasing population. Thus, proteins from cost-effective and non-conventional sources have gained more attention for livestock production. Therefore, the present study investigated the effects of partial replacement of soybean meal with 5% *C. vulgaris* or 5% *C. vulgaris* with lysozyme on the growth performance, diarrhea rate, immune function, plasma biochemical parameters, and colonic microbiota and metabolites of weaned pigs. The findings showed that *C. vulgaris* supplementation reduced diarrhea rate and enhanced plasma biochemical parameters, whereas decreased IgM secretion and SCFA production. On the other hand, *C. vulgaris* with lysozyme supplementation showed an increased diarrhea rate, improved immune function by increasing immunoglobulin secretion and immuno-cytokines production, and increased colonic beneficial microbiome abundances and metabolites production in weaned Xiangcun black pigs compared with the CHV group.

Piglet diarrhea during the early stage of life is one of the major causes that can seriously affect the outcome of extensive animal production (15). Changes in diet structure during weaning are closely associated with piglet diarrhea and are accompanied by several health complications such as dehydration, growth retardation, and mortality of piglets (16). Consistent with a previous study (17), our results showed that soybean meal partially replaced by 5% *C. vulgaris* with or without lysozyme had no impact on the BW, ADG, ADFI, and F/G of weaned piglets. Moreover, several previous studies aimed to enhance growth performance and ameliorate post-weaning stress using *C. vulgaris* (≤1% or 385 mg/kg) as feed supplements and found no significant differences in ADFI, ADG, and feed conversion ratio in weaned piglets (18, 19). Interestingly, soybean meal partially replaced by *C. vulgaris* reduced diarrhea rate of piglets, while *C. vulgaris* supplementation with lysozyme increased the diarrhea rate of weaned piglets in the present study. The increased diarrhea rate in the LYSO group might be due to the fact that lysozyme may be involved in dissolving the cell wall of *C. vulgaris* to release additional nutrients (such as proteins) in the small intestine (7), causing a higher diarrhea rate when *C. vulgaris* is supplemented with lysozyme. However, further research is needed to understand the underlying mechanisms.

Plasma biochemical parameters have been increasingly used as physiological conditions as they represent valuable information about the host health condition of animals (20). It has been previously reported that certain microalgae species (such as *Porphyra tenera* and *Laminaria digitata*) enhance lipid metabolism through increasing lipoprotein profiles (such as lower LDL-C and higher HDL-C and lower cholesterol-enriched VLDL-C) in serum of a rat model, especially in individuals with hyperlipidemia (21). Microalgae are mainly composed of different bioactive compounds, such as polysaccharides, fibers, and lipids (i.e., omega-3 fatty acids), which are associated with lipid metabolism by lowering TG and LDL-C and increasing HDL-C levels (22, 23). Concerning the lipid metabolism biomarkers, different effects were observed by *C. vulgaris* and lysozyme supplementation. In the present study, soybean meal partially replaced by 5% *C. vulgaris* displayed an increasing trend in the plasma ALB and ALP levels compared with the pigs supplemented with the basal diet, indicating enhanced the immunity of piglets. Previous studies reported that higher level of ALB in serum can enhance the immunity and play protective role for immunoglobulins (24). Furthermore, ALP can contribute to the promotion of the differentiation of T lymphocytes and secretion of immunoglobulin to affect the humoral immune response (25). Additionally, although 5% *C. vulgaris* supplementation with lysozyme increased the TC level, which is in agreement with a previous study (26), as cholesterol could be partially obtained from the diet either by consumption of animal-derived products or from de-novo biosynthesis in the liver. Furthermore, the increased TC was counterbalanced by a significant increase in plasma HDL-C level in the CHV and LYSO groups, apparently leading to healthy cardiovascular functions (27). Moreover, reverse cholesterol transport can remove excess cholesterol from peripheral tissues and deliver it to the liver, while it is possibly redistributed to other tissues or removed from the key organ, as HDL-C is the main lipoprotein associated with this process. These findings indicate the beneficial effects of dietary *C. vulgaris* and lysozyme on fatty liver protection, as well as fatty liver-associated different metabolic disorders like obesity, diabetes, and hyperlipidemia (28).

Research evidence indicated that supplementing animal diets with microalgae has exhibited immune-enhancing functions (27). Pro- and anti-inflammatory cytokines dynamically regulate the immune function of weaned pigs. For instance, higher levels of pro-inflammatory cytokines, including IL-1β, IL-6, and TNF-*α*, lead to immune dysregulation, while anti-inflammatory cytokines (such as IL-10) can reduce inflammation (29). Previous studies have demonstrated that microalgae-based (such as *Ulva lactua*, *A. platensis*, and *C. vulgaris*) diets have significant anti-inflammatory effects in weaned pigs by regulating several immuno-cytokine profiles (19, 27). Consistent with these studies, our results showed that IL-6 level was lower, whereas IL-10 level was higher in jejunum of the LYSO group, indicating the potentiality of anti-inflammatory and immunosuppressive effects that regulated normal tissue homeostasis (30, 31). Additionally, the CHV group had a reduced the TNF-α level in the ileum of piglets. Moreover, TNF-α level in the ileum was also positively correlated with beneficial bacterial taxa, such as *Lactobacillus* and *Blautia_A*, and it was negatively correlated with *Escherichia* and *Streptococcus*. The immune structure of the jejunum, such as the higher density of immune cells and T lymphoid tissues, facilitates a more robust interaction between the lysozyme and immune cells, thus promoting the release and regulation of immuno-cytokines (32). On the other hand, the microbiome population differs between the jejunum and ileum, of which the jejunum has a comparatively lower bacterial load, while polysaccharides and bioactive compounds present in microalgae could enhance beneficial bacteria and further strengthen the immune response (33, 34). These might be the possible reasons that the anti-inflammatory and immunosuppressive repose of *C. vulgaris* with lysozyme was higher in the jejunum, while *C. vulgaris* without lysozyme exhibited a higher response in the ileum of piglets. These findings indicate that soybean meal parially replaced by 5% *C. vulgaris* without lysozyme may also partially contribute to the reduction of inflammation in weaned pigs.

Accordingly, the present study also evaluated the effects of *C. vulgaris* and lysozyme on the plasma immunoglobulins of weaned pigs. Although there were no significant differences in the plasma IgA and IgG concentrations, but plasma IgM was significantly lower in the CHV group compared with the other two groups. Moreover, plasma IgM was positively correlated with colonic *Lactobacillus* and *Schwartzia* abundances. These findings are consistent with a previous study by Matrins et al. (26), who reported that the IgM level could rise in the short-term and then begin to drop as the secretion of other immunoglobulins increases, thereby providing long-term protection for the immune organs. Overall, our findings suggest that soybean meal partially replaced by *C. vulgaris* exhibited lower immunity in piglets, while *C. vulgaris* supplemented with lysozyme recovered that effect. This indicates that *C. vulgaris* supplementation with lysozyme has significantly better immune effects than *C. vulgaris* alone.

The intestinal microbiota and microbiota-derived metabolites play crucial roles in various physiological and pathophysiological processes to regulate the gastrointestinal and immune function of the host. Microbial diversity indicators are commonly known as the representative of the gut ecosystem stability and the health of the host. The lower intestinal microbiota diversity indicates the dysbiosis of microbiota, suggesting that the host is affected by pathogen or pathogenic bacteria (35). In the present study, dietary *C. vulgaris* or *C. vulgaris* supplemented with lysozyme had no impact on the colonic microbiota diversity of weaned pigs. However, *C. vulgaris* supplementation without lysozyme showed an increasing trend in the Simpson index, indicating partial beneficial effects on maintaining the intestinal homeostasis of weaned pigs (36).

At the phylum level, our findings revealed that the most abundant bacterial phyla in all groups were Firmicutes, Bacteroidota, and Actinobacteria, which are in agreement with previous studies (17, 37). The taxonomic difference indicated that Firmicutes abundance was decreased, while Desulfobacterota abundance was increased in the CHV and LYSO groups compared with the CON group. Firmicutes are mainly associated with energy absorption from nutrient substances and SCFA production by the fermentative metabolism and degradation of carbon sources, proteins, and amino acids (38). Therefore, the decreased Firmicutes abundance in the CHV and LYSO groups affected the SCFA production, as evidenced by lower SFCA concentrations in the colon of weaned pigs in the present study. This decrease in both Firmicutes and SCFA (including acetate, butyrate, propionate, and valerate) indicates the presence of lower levels of fermentable carbohydrates in the colon of weaned pigs, which may be due to the recalcitrant wall of *C. vulgaris* that inhibited fermentation of carbohydrates in the digestive compartment. Previously, it has also been reported that diets consisting of insoluble dietary fiber affected the fermentation and the production and absorption of SCFA in the large intestine of pigs (39). In the present study, *C. vulgaris* supplementation with lysozyme increased Actinobacteriota abundance in the colon of weaned piglets. Several studies have found that Actinobacteriota is related to the production of antibiotics and immunomodulatory metabolites, which possess intestinal inflammation and immunity (40, 41), as evidenced by reduced IL-6 and elevated IL-10 levels in the jejunum of the LYSO group in the present study.

At the genus level, microbiome analyses showed that *Megasphaera_A* abundance was decreased and *Phascolarctobacterium_A* abundance was increased in the CHV and LYSO groups compared with the CON group. The higher abundance of *Phascolarctobacterium* leads to the promotion of the growth of symbiotic bacteria in the intestine to protect against intestinal diseases (42). A previous study indicated that *Megasphaera* is a potential bacterial genus that converts lactate to acetate, butyrate, propionate, and valerate and plays essential roles in gut health (43). This might be one of the possible reasons that the SCFA concentrations were lower in the CHV and LYSO groups.

The correlation analysis also revealed that colonic *Megasphaera_A* abundance was positively correlated with propionate and valerate concentrations. Furthermore, *C. vulgaris* with lysozyme supplementation increased the abundances of *Faecalibacterium* and *Anaerovibrio* in the colon. These findings are consistent with LEfSe results, which revealed that *Faecalibacterium* and *Anaerovibrio* were enriched in the LYSO group. Research evidence indicated that reduced *Faecalibacterium* abundance is associated with certain disorders such as celiac disease, chronic diarrhea, and inflammatory bowel diseases (44). *Anaerovibrio* are considered a group of lipid-degrading bacteria, which can inhibit the oxidation reactions during gastrointestinal digestion and protect against gastrointestinal inflammation (42). Taken together, although diets supplemented with *C. vulgaris* without lysozyme displayed causal effects of several pathogenic bacteria associated with SCFA production due to the presence of the recalcitrant wall of microalgae while *C. vulgaris* supplemented with lysozyme had beneficial effects in some contexts to enhance the plasma biochemical parameters and immune function of weaned pigs.

## 5 Conclusion

In this study, we found that partially replacing soybean meal with *C. vulgaris* reduces the diarrhea rate without affecting the growth performance of weaned piglets. Additionally, soybean meal replaced with *C. vulgaris* and lysozyme partially improves intestinal immunity by reducing pro-inflammatory and enhancing anti-inflammatory cytokines in the jejunum of weaned pigs. Moreover, *C. vulgaris* with lysozyme enhances several beneficial gut bacteria and metabolites related to lipid degradation and host immunity in comparison to *C. vulgaris* alone. Nevertheless, partially replacing soybean meal with *C. vulgaris* and lysozyme could be a promising non-conventional dietary supplemental approach for cost-effective swine production.

## Data Availability

The datasets presented in this study can be found in online repositories. The names of the repository/repositories and accessions number(s) can be found at the Science Data Bank under accession number https://doi.org/10.57760/sciencedb.16959.
